# Noninvasive Follicular Thyroid Neoplasm with Papillary-like Nuclear Features (NIFTP): Tumour Entity with a Short History. A Review on Challenges in Our Microscopes, Molecular and Ultrasonographic Profile

**DOI:** 10.3390/diagnostics12020250

**Published:** 2022-01-20

**Authors:** Ivana Kholová, Elina Haaga, Jaroslav Ludvik, David Kalfert, Marie Ludvikova

**Affiliations:** 1 Pathology, Fimlab Laboratories, Arvo Ylpön Katu 4, 33520 Tampere, Finland; elina.haaga@tuni.fi; 2 Faculty of Medicine and Health Technology, Tampere University, Arvo Ylpön Katu 34, 33520 Tampere, Finland; 3 Department of Imaging Methods, University Hospital Pilsen, Faculty of Medicine in Pilsen, Charles University, Alej Svobody 80, 30460 Pilsen, Czech Republic; ludvikj@fnplzen.cz; 4 Department of Otorhinolaryngology and Head and Neck Surgery, First Faculty of Medicine, University Hospital Motol, Charles University, 15006 Prague, Czech Republic; david.kalfert@fnmotol.cz; 5 Department of Biology, Faculty of Medicine in Pilsen, Charles University, 32300 Pilsen, Czech Republic; ludvikova.m@email.cz

**Keywords:** Noninvasive Follicular Thyroid Neoplasm with Papillary-like Nuclear Features (NIFTP), thyroid gland, thyroid papillary carcinoma, histopathology, cytology, ultrasound, molecular diagnosis, The Bethesda System for Reporting Thyroid Cytopathology

## Abstract

Since Noninvasive Follicular Thyroid Neoplasm with Papillary-like Nuclear Features (NIFTP) was introduced as a new thyroid tumour entity, many studies, and meta-analyses on diagnosing NIFTP have been published. NIFTP-revised histopathological criteria emerged in 2018. NIFTP is defined as a histological entity and its diagnosis requires a careful histological examination. Its molecular profile is similar to follicular-like tumours. Ultrasound features are unable to differentiate NIFTP. NIFTP is not a cytological diagnosis, but it influences the risk of malignancy in several categories of The Bethesda System for Reporting Thyroid Cytopathology terminology.

## 1. NIFTP: Historical Overview

In the 1950s and 1960s, thyroid tumour diagnosing was based on the following architecture: papillae were the histopathological mainstay for papillary carcinoma (PTC) and follicles for follicular adenoma/carcinoma [1]. In 1960, Lindsay recognized, for the first time, the nuclear features of papillary carcinoma in follicular-patterned tumours [2], but it was later, in 1977, when Chem and Rosai defined the follicular variant of papillary carcinoma (FVPTC) [3]. In 2000, the proposal of “Well-Differentiated Tumour of Uncertain Malignant Potential” (WDT-UMP) characterized as an encapsulated tumour composed of well-differentiated follicular cells with questionable papillary carcinoma nuclear features and no-invasion was published, but this was not widely accepted [1,4]. The molecular analyses and positive clinical outcome further supported the fact that encapsulated FVPTC is an entity distinct form classical PTC and other variants [1]. Finally, an international multidisciplinary Prof. Yuri Nikiforov led expert panel group including pathologists, clinicians and patients issued a diagnostic consensus on a new entity Noninvasive Follicular Thyroid Neoplasm with Papillary-like Nuclear Features (NIFTP) based on the analysis of 268 cases [5]. In 2017, the entity was implemented in a new edition of a WHO classification of tumours of endocrine organs which was also accepted by the American Thyroid Association in their treatment recommendations [6,7].

**Figure 1:**
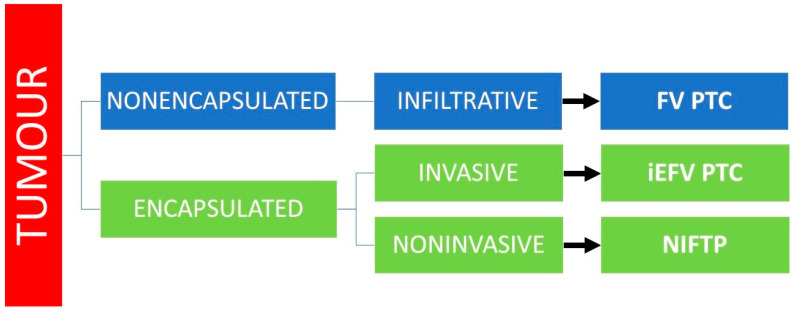
Classification of follicular—patterned thyroid tumours with papillary-like nuclear feature. (Abbreviations: FVPTC, follicular variant of papillary thyroid carcinoma; iEFVPTC, invasive encapsulated follicular variant of papillary thyroid carcinoma; NIFTP, noninvasive follicular thyroid neoplasm with papillary–like nuclear features).

A schematic explanation of classification of follicular-patterned thyroid tumours with papillary-like nuclear feature is shown in Figure 1.

The main reason for a new nomenclature was the aim to avoid the word carcinoma and both the overdiagnosis and overtreatment of tumors with indolent biological behavior and a good prognosis [5]. Financial, emotional, and even the psychiatric burden of thyroid cancer diagnosis is acknowledged [8]. Nevertheless, Parente et al. found five cases (4.9%) that presented with lymph node metastasis and one case with a distant metastasis in their cohort of 102 NIFTPs, that were diagnosed using originally proposed criteria [9]. 

Moreover, the other authors also reported, in their NIFTP studies, several cases with lymph node metastases, highlighting the necessity for stricter diagnostic criteria [10,11].

## 2. NIFTP: Histopathology

Originally, the reclassification of noninvasive, encapsulated FVPTC into NIFTP was based on international series of 109 cases re-evaluated by 24 thyroid pathology experts. Consensus diagnostic criteria were divided into major and minor features and exclusion criteria [5].

**Figure 2:**
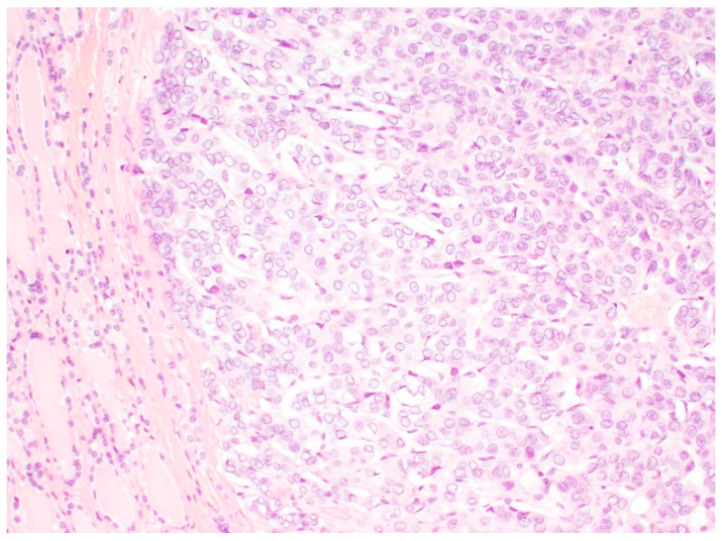
Encapsulated thyroid tumour formed by microfollicles fulfilling the histopathological criteria of NIFTP. Original magnification 100×.

The histopathological diagnostic criteria of NIFTP were revised in 2018. The diagnosis of NIFTP requires the primary criteria defined as encapsulation or clear demarcation of the lesion with no vascular or capsular invasion, follicular growth pattern with no well-formed papillae, no psammoma bodies and less than 30% of solid, trabecular, or insular growth pattern. The assessment of nuclear size and shape, nuclear membrane irregularities and chromatin characteristics concluded as nuclear score of 2 or 3 is required. Tumour necrosis and high mitotic activity are not acceptable (Figure 2). The secondary criteria are not required but may be helpful. The secondary criteria include a lack of the *BRAF^V600E^* mutation, *BRAF^V600E^*-like mutations or other high-risk mutations diagnosed by molecular assays or immunohistochemistry [5,12,13].

The fourth edition of the WHO Classification of Tumours of Endocrine Organs defined NIFTP as follows “non-invasive follicular thyroid neoplasm with papillary-like nuclear features (NIFTP) is a non-invasive neoplasm of thyroid follicular cells with a follicular growth pattern and nuclear features of papillary thyroid carcinoma (PTC) that has an extremely low malignant potential” [6]. The word “carcinoma” is replaced by the word “neoplasm” as a real-life adoption of a new terminology, rather than as an academic etymology exercise [8]. Of note, the press reported NIFTP as a benign tumour, despite it was seen as a preinvasive neoplasm best compared to in situ carcinoma of the breast [14].

A thorough pathological work-up is needed. The overall microscopic examination to assess the entire capsule and the absence of true papillae, psammoma bodies, exclusive growth pattern and necrosis is warranted [14,15]. NIFTP does not require formal staging [15]. Interestingly, several cases of NIFTP with metastases were recently published. Most of them showed lymph node metastases, though two NIFTP were associated with distant bone and pulmonary metastases as well [9,11,16,17]. If all these NIFTP cases met the revised morphological diagnostic criteria, these findings illustrate very well that despite the prognosis of NIFTP being previously declared as excellent, the risk of adverse outcomes due to metastases is not negligible. Therefore, the continuing follow-up of NIFTP patients is necessary to avoid the unwanted progression of the disease [16,17]. Open questions to be answered concern micro-NIFTP, oncocytic NIFTP, multifocal NIFTP, and long-term prognostic implications [14].

## 3. NIFTP: Epidemiology

The original rate of 18.6%, among 3400 PTC cases, published in a seminal paper [5] has been shown to be higher than average in recent analyses. The recent meta-analysis showed the worldwide NIFTP incidence to be 6%, with an Asian vs. Western discrepancy [18]. The Asian continent was represented by 28 institutions from 9 countries and the incidence varied from 0.4% in China to 14.4% in India with 2.1% NIFTP Asian rate on average [18]. The reasons for discrepancy are multifactorial, and may be as follows: racial and ethnical background and mutation profile represent biological factors [18], but the threshold for diagnosing PTC nuclear features was shown to differ in American vs. Japanese and Korean series [18,19,20,21,22]. In addition, data collection from surgical or cytological databases played a role [18]. Furthermore, in 2018 NIFTP criteria were refined, leading to a decline of almost 2.5 in NIFTP incidence [18]. The entire capsule assessment was suggested to enhance the detection of capsular invasion [18]. In contrast, active surveillance without surgical resection is the preferred practice in Asia, which can also contribute to biases [23]. Interestingly, in addition to a significant reduction of FVPTC, a modest reduction of PTC prevalence is expected following the introduction of NIFTP into practice [24].

## 4. NIFTP: Molecular Studies

The Cancer Genome Atlas (TCGA) data showed that differentiated thyroid carcinoma is a tumour with one of the lowest tumour mutational burdens. A single driver gene alteration, such as *BRAF* and *RAS* mutations, or fusion genes, is involved in the development of neoplasm constitutive of the MAPK (mitogen activated protein kinase) signaling pathway [25]. The additional genetic alterations may modify the biological behavior of thyroid tumours, for instance *TERT* reactivation prevents telomere shortening and has been associated with a poor outcome of differentiated thyroid cancer [26]. The results of molecular genetic studies of thyroid neoplasms can be translated into clinical practice as an ancillary tool for diagnosis and prognosis evaluation.

After the establishment of NIFTP as a new thyroid tumour entity in 2016, researchers aimed to determine its molecular profile. Researchers focused on molecular, genetic and/or immunohistochemical testing with *BRAF^V600E^* mutation and other high-risk mutations (for example *TERT* promoter mutation) that were not fully compatible with the diagnosis of NIFTP [12,13]. With respect to original morphological criterion “less than 1% papillae in NIFTP”, molecular differences between “NIFTP with papillae” and “NIFTP without papillae at all” were described. Particularly, the former feature was associated with a *BRAF^V600E^* mutation that may be responsible for a worse clinical prognosis and recurrence [11,27]. This fact contributed to the critical revision and establishment of the stricter diagnostic criteria for NIFTP in 2018 [12,13]. As mentioned earlier, NIFTP diagnostic criteria are histological and do not require the secondary diagnostic criteria. Nevertheless, the additional molecular criteria are considered helpful [12].

Molecular analysis can serve as an additional method in a subset cases of NIFTP, for instance, in tumours larger than 4cm in size when the entire parenchyma is not sampled [28] or in nodules that are difficult to classify based on cytologic features alone [29].

In the light of molecular genetic characteristics of NIFTP, the published results are conflicting. NIFTPs are primarily associated with activating mutations of *RAS* genes (mutation of *NRAS* gene predominates above *HRAS* and *KRAS* genes mutations) found in 30–67% of cases [30,31,32]. This molecular feature is similar to follicular-patterned thyroid tumours. Less commonly NIFPTs harbor *PAX8-PARG* gamma and *THADA* rearrangements [30,33,34]. No *RET/PTC* rearrangements were described in NIFTP. None of the mutations detected in NIFTPs are pathognomonic of this entity [35]. Variability and overlapping of genetic profiles of NIFTP were presented by several authors. Pool et al. used commercially available molecular tests Afirma and Thyroseq and revealed the molecular diversity of NIFTPs with cases grouped into “*BRAF*-like”, “*RAS*-family-like”, and “*THADA*-like” [33]. The most common variant of *BRAF* mutation in NIFTP is *BRAF^K601E^* mutation associated with better outcome comparing with *BRAF^V600E^* one [36,37,38]. Nevertheless, *BRAF^V600E^* mutation was also sporadically reported, including two Korean studies with the frequency 8–28% in NIFTP [10,27,39]. Less common molecular abnormalities in tumours featuring NIFTP were also documented, such as *TERT* promoter (*TERTp*) mutation and *ETV6-NTRK3* fusion [40]. Although these high-risk mutations were described as indicators of poor prognosis in differentiated carcinomas, no prognostic association was revealed between *TERTp* mutation and NIFTP [41]. Because such mutations represent the exclusion criterion for NIFTP diagnosis, an extensive search for morphologic exclusion criteria should be conducted [12].

Recently, Kuchareczko et al. compared NIFTPs diagnosed according to both initial (2016) and revised (2018) diagnostic criteria in their study. They found a *BRAF^V600E^* mutation in the former NIFTP diagnostic group only, but not in revised NIFTP cases. However, other high-risk mutations (e.g., *TERT* promoter mutation and *TP53* mutation) were found in both groups of NIFTP [42]. In accordance with this study, Sohn et al. also detected *BRAF^V600E^* mutation in invasive encapsulated FVPTC (iEFVPTC) (9.1%) and infiltrative FVPTC (37.5%), but in none of the 10 NIFTP in their series, which were classified according to revised criteria [43]. If high-risk mutations are revealed in NIFTP cases, such tumours require more intensive search for invasive feature and papillae as well as the long-term follow-up [12,42]. Any high-risk mutations constitute the exclusion criterion for NIFTP diagnosis [12]. The association of NIFTP metastatic phenotype with high-risk mutation seems to be one of possible explanation of published cases [9,11,16,17]. Therefore, further studies including molecular profiles of these metastatic cases are required.

Although NIFTP was considered to be a variant of papillary carcinoma before its reclassification, its molecular profile is better matched to follicular tumours, namely follicular adenoma, follicular carcinoma, and invasive encapsulated FVPTC (iEFVPTC). According to some authors, NIFTP is molecularly distinct both from infiltrative FVPTC and from classical PTC, but only few molecular differences were found between NIFTP and iEFVPTC [43,44]. NIFTP and iEFVPTC show shift from *BRAF^V600E^*-like to *RAS*-like molecular signature, but an overlapping molecular profile with various papillary and follicular neoplasms exists [33,45]. NIFTP molecular diversity and overlaps could also contribute to the discrepancy among studies [12].

Taken together, NIFTP molecular profiles are closer matched to follicular-pattern thyroid tumours, which is a predominant activating mutation of the *RAS* gene and other less common genetic alterations, such as *PPARG* and *THADA* fusions and *BRAF^K600E^* mutations, which are mainly detected [33]. However, to date, a NIFTP-specific molecular profile has not been established, therefore strict morphological criteria for NIFTP are fundamental in diagnosis of this lesion and molecular indicators may aid only to avoid the overdiagnosis or to neglect lesions with more aggressive behavior [45].

In addition to molecular genetic testing, the epigenetic analysis of thyroid neoplasms including newly established NIFTP lesion has been lately performed. Attention was paid to non-coding RNA, namely on microRNA (miRNA; miR) [46,47,48]. MiRNA molecules play a crucial role for cell differentiation, migration, and invasion by regulating target genes of various signaling pathway [49,50]. Various studies focused predominantly on the expression profiles of selected microRNA in subtypes of PTC and NIFTP [46,47,48]. Jahanbani et al. demonstrated miR-222-3p and miR-146-5p to be useful discriminatory markers between follicular variant of PTC and NIFTP [46]. A different study presented by Park et al. identified three miRNA markers (miR-21; miR-136, and miR-127) with low expression levels in NIFTP in contrary to their high level of expression of these miRNAs in differentiated thyroid cancers, especially in follicular and other variants of PTC with metastases and a more aggressive outcome [47]. Borelli et al. introduced a whole panel of miRNAs that showed differential expression between follicular adenomas, NIFTP and iFVPTC. Thus, some of the presented miRNAs may be promising discriminatory markers [48]. In addition to miRNA, the potential emerging diagnostic role of long non-coding RNA in thyroid lesions is emerging too [51].

## 5. NIFTP: Ultrasonographic Studies

On the ultrasound (US) level, the NIFTP is an oval, well-circumscribed nodule with well detected margins and variable echogenicity, which may be hypoechoic, isoechogenic or heterogeneous [52,53]. However, echogenicity feature assignment is subject to inter-observer variation with very low agreement [54]. A US grading of risk of malignancy of thyroid nodules was included in the guidelines on thyroid cancer management that was introduced by the British Thyroid Association (BTA) in 2014 [55]. A new standardized system for the classification and management of thyroid nodules based on US features (e.g., the Thyroid Imaging Reporting and Data System, TI-RADS) was created in 2017 by The American College of Radiology (ACR). The aim of the system was to improve thyroid-nodule management, based on an US assessment across five categories–composition, echogenicity, shape, margin, and echogenic foci- and subsequent five suspicion levels. Features of each category are associated with 0–3 points that determine for every nodule its suspicion level, which range from TR1 (benign) to TR5 (highly suspicious) [56,57]. In addition to ACR-TI-RADS system, similar ultrasound risk–stratification systems were established by Korean Society of Thyroid Radiology (K-TI-RADS), American Thyroid Association (ATA-TI-RADS) and by the American Association of Clinical Endocrinologists, the American College of Endocrinologists and Assoziatione Medici Endocrinologi (AACE/ACE/AME-TI-RADS) to identify high-risk thyroid nodules and evaluate the need for fine-needle aspiration [58,59]. Recently, a scoring system based on artificial intelligence (AI), known as AI-TI-RADS, was proposed as an attempt to refine some previous TI-RADS systems [55]. The ACR-TI-RADS classification system seems to be the best for the determination of cytologically high-risk nodules according to some authors [59,60]. However, there is still no generally accepted consensus on the most efficient TI-RADS stratification model, since the results of the studies are not straightforward. A variety of US grading systems have comparable diagnostic performance, namely, high sensitivity for predicting thyroid nodule malignancy while there are discrepancies in other parameters as highlighted by Watkins et al. [55].

Several studies, recently, have focused on the US diagnosis of NIFTP as well as on the differentiation between NIFTP and both encapsulated and infiltrative forms of FVPTC [61,62,63,64,65]. These studies evaluated thyroid nodules risk-stratification using different guidelines. They concluded that NIFTP nodules were not highly suspicious in the US criteria (TR5 on ACR score) while TR5 was detected in EFVPTC with low frequency (from 8% to 14.6%) and in IFVPTC with high frequency (range 64–65.3%) [61,62,63,65,66,67]. Rosario et al., in agreement with ACR-TI-RADS, confirmed that the majority of NIFTP were assigned as TR3 (mildly suspicious) and TR4 (moderately suspicious) [68]. A highly suspicious ultrasound (TR5) seems, therefore, to be a good marker against diagnosis of NIFTP before surgery.

The other studies attempted to compare US characteristics between NIFTP and similar non-NIFTP tumours (classical and follicular variants of PTC, follicular adenoma, etc.). Significant US differences between both groups were confirmed regarding predominantly echogenicity, margins, microcalcifications, and Doppler flow pattern. Prevalent non-suspect US NIFTP characteristics encompass iso- and hyperechoic features, regular smooth margins, perinodular and intranodular Doppler flow pattern and the absence of microcalcifications [53,64,67]. Similar non-suspect US features were described by Brandler et al. in follicular adenomas [35]. Conversely, Brandler et al. described no significant differences in echogenicity between NIFTP and PTC [35]. Significant differences were confirmed in the US profile of NIFTP and iFVPTC and classical PTC, while differentiation between NIFTP and invasive EFVPTC is more difficult because of the overlapping US characteristics [62,64,68]. Doppler US in NIFTP and iEFVPTC is inconclusive, showing no significant differences in intranodal vascularity, which is indicative of a malignancy [62,64,68,69].

A robust multicenter study of 257 thyroid nodules with indeterminate fine needle aspiration cytology, US pattern (based on American Thyroid Association [ATA] and The American College of Radiology Thyroid Image Reporting and Data System [TI-RADS] US risk-stratification systems) and ThyroSeq v3 molecular testing to refine cancer/NIFTP probability in thyroid nodules was recently published. They concluded that no statistically significant advantages for the use of US were found when compared to molecular testing in terms of their discrimination between benign versus cancer/NIFTP cytologically indeterminate nodules [70].

In general, US is the pivotal tool used to reveal and characterize thyroid nodules. However, the conventional US imaging techniques are limited in several aspects, namely, in inter-observer variability and spatial resolution. Therefore, the introduction of new high-resolution imaging technologies such as contrast-enhanced US (CEUS) and US-elastography seem to be the promising diagnostic modalities to assess even better thyroid nodules. CEUS could improve the evaluation of the vascularity and perfusion of thyroid tumours, that is, the character of malignant tumours, while US-elastography detects the tissue stiffness due to fibrosis [71,72]. The combined elastography and traditional US approach has proved its utility in cytologically indeterminate thyroid nodules [73]. One of many elastographic techniques, qualitative strain elastography, was shown to be a promising diagnostic tool for the assessment of thyroid nodules with indeterminate cytology. Based on recent studies, qualitative strain elastography can act as a potential new parameter of the TI-RADS [74]. Moreover, the combined use of classical US diagnostic technique, elastography and volumetric Doppler examination can improve pre-surgical risk assessment of intermediate thyroid nodules and improve further management of these cases [75]. At moment nevertheless, we are not aware of any study focusing on the detection of NIFTP nodule by listed new imaging technologies.

An US analysis of thyroid nodules may lead to the detection for NIFTP; however, US is not able to fully differentiate between NIFTP and iEFVPTC. Therefore, the attempts of preoperative identifications of NIFTP should always combine ultrasound with fine needle aspiration biopsy and molecular analysis. Nevertheless, the final diagnosis is based on the histological evaluation of the entire thyroid nodule.

## 6. NIFTP: Cytopathology and Summary of Cytological Studies

NIFTP is not a cytological diagnosis. NIFTP is defined as a histological entity and its diagnosis requires a careful histological examination as referenced above. After NIFTP was introduced as a new entity, many studies on the cytology of NIFTP have been published. Several studies on cytological diagnostic features showed that features related to NIFTP differ mainly from PTC cytopathology and to lesser extent from FVPTC cytopathology [67,76,77,78,79,80,81,82,83,84,85,86,87,88,89,90,91,92].

In summary, NIFTP showed architecturally less papillae, psammoma bodies and giant cells than PTC in cytological specimens. On the other hand, microfollicles were more frequent in NIFTP than in PTC. On nuclear level, chromatin clearing, contour irregularities, enlargement, grooves, and pseudoinclusions were statistically significantly more expressed in PTC cases than in NIFTP cases [67,76,77,78,79,80,81,82,83,84,85,86,87,88,89,90,91,92,93].

NIFTP revealed similar cytomorphological scores to FVPTC in many of its features. However, statistically significant differences were revealed between NIFTP and FVPTC as shown by a recent meta-analysis. NIFTP was more likely to have microfollicles than FVPTC. On contrary, FVPTC showed papillae, giant cells, nuclear elongation, enlargement, grooves and pseudoinclusions more frequently than NIFTP. Importantly, the presence of chromatin clearing, contour irregularities, crowding and psammoma bodies revealed no statistically significant differences between NIFTP and FVPTC cases [67,76,77,78,79,80,81,82,83,84,85,86,87,88,89,90,91,92,93].

Despite the cytomorphological differences summarized above, NIFTP is not a cytological diagnosis and final diagnosis requires histological assessment.

## 7. NIFTP: Bethesda Categories and ROM

Fine needle aspirations from the thyroid gland are categorically classified by a six-tiered system. The Bethesda System for Reporting Thyroid Cytopathology (TBSRTC) was introduced as the first non-gynecological organ-specific cytopathology terminology in 2010 [94], with the 2nd edition from 2017 taking into the account the introduction of NIFTP entity [95].

In the majority of published series, NIFTP cases were placed in intermediate categories of TBSRTC, whereby 29.8% of cases are in atypia of undetermined significance/follicular lesion of undetermined significance (AUS/FLUS), 28.0% in follicular neoplasm (FN) and 21.2% in suspicious for malignancy (SM) categories. However, 1.8% of the samples were categorized as non-diagnostic (ND) category, 10.2% as benign category and 8.4% as malignant category according to a meta-analysis conducted by Haaga et al. [93]. Ruanpeng et al. and Bongiovanni et al. showed, in their meta-analyses, similar NIFTP cases distribution in TBSRTC categories: 3.6% and 3% cases were placed in ND category, 10.0% and 10% in benign category, 34.2% and 30% in AUS/FLUS category, 22.7% and 21% in FN category, 22.4% and 24% in SM category and 7.5% and 8% in malignant category [24,96].

Accordingly, the 2nd edition [95,97] revised the calculated risks of malignancy (ROM) in TBSRTC categories, as non-malignant NIFTP status affects ROM in majority of categories. In summary, ROMs decreased in the intermediate categories that are characterized by the high prevalence of histologically diagnosed FVPTC and NIFTP cases [97,98,99,100]. In a recent meta-analysis, pooled risk differences of ROM were performed in each TBSRTC category, independently resulting in the following reductions: 2.4% (I2 was 0%) in ND, 2.7% (I2 was 2%) in benign, 8.2% (I2 was 43%) in AUS/FLUS, 8.2% (I2 was 53%) in FN, 7.3% (I2 was 89%) in SM, and 1.1% (I2 was 45%) in malignant category when NIFTP was reclassified [93]. Similarly, Layfield et al. conducted a meta-analysis of three published studies and their own data in 2017, which showed significantly lower ROM in all TBSRTC categories except for the ND category [101]. On the other hand, Bongiovanni et al. only showed a reduction in the ROM for SM and malignant categories by 14% and 3%, respectively [102].

Interestingly, the risk differences of ROM were reduced by 2.6% (I2 was 0%) in ND, 2.7% (I2 was 0%) in benign, 4.7% (I2 was 0%) in AUS/FLUS, 6.0% (I2 was 45%) in FN, 1.8% (I2 was 29%) in SM and 0.3% (I2 was 28%) in malignant category in Asian countries in comparison to non-Asian countries with corresponding figures being 1.9% (I2 was 0%) in ND, 1.9% (I2 was 5%) in benign, 9.7% (I2 was 52%) in AUS/FLUS, 9.1% (I2 was 27%) in FN, 14.7% (I2 was 73%) in SM and 1.7% (I2 was 0%) in malignant category [93]. The differences between the Asian and non-Asian series were statistically significant in AUS/FLUS (*p* = 0.023), FN (*p* = 0.001) and malignant (*p* = 0.008) categories, as revealed in meta-analysis by Haaga et al. [93]. The thyroid pathology practice specificities and ethnic and mutational background contribute to the differences in Asian and non-Asian series [23].

When NIFTP incidence was taken into the account, with a 5% NIFTP incidence threshold, statistically significant differences were observed in AUS/FLUS and FN TBSRTC categories. In the AUS/FLUS category, the mean decrease in ROM was 13.5% in studies with >5% incidence and 5.1% in studies with <5% incidence (*p* = 0.036). In FN category, the mean decrease in ROM was 17.2% and 5.6%, respectively (*p* = 0.005) [93].

## 8. NIFTP: Prognosis and Clinical Work-Up

NIFTP was defined as a “a non-invasive neoplasm of thyroid follicular cells with a follicular growth pattern and nuclear features of papillary thyroid carcinoma (PTC) that has an extremely low malignant potential” in the fourth edition of WHO Classification of Tumours of Endocrine Organs [6]. The definition and terminology switch from carcinoma to neoplasm [8] also indicates indolent biological behavior and a good prognosis [5,6]. The updated and stricter histopathological criteria [12] will enhance good NIFTP prognoses, as possible lesions with adverse prognosis will be excluded. Namely, the presence of true papillae, psammoma bodies, solid, trabecular, or insular growth pattern and necrosis are histopathological features with dismal prognostic potential [14,15]. The strict use of updated histopathological criteria will preserve the low biological potential of NIFTP to recur or form metastases. Nevertheless, long term data are yet to be reported [14]. A French-Swiss multi-institutional retrospective study of 363 patients with a median follow-up of 5 years did not report any lymph node metastases nor recurrency in the cohort [103]. A Canadian series of 102 NIFTP cases with a mean follow up of 5.7 years revealed five lymph node metastases and one distant metastasis, forming 6% of cases with malignant behavior, which was nevertheless performed with original criteria [9].

According to the literature on noninvasive encapsulated FVPTC, cases reclassified into NIFTP were treated by total thyroidectomy often followed by radioactive iodine treatment [104,105]. Current knowledge of the biological potential of NIFTP will allow for a more conservative treatment in the form of a lobectomy [104]. Furthermore, the Asian approach is far more conservative and even diagnostic lobectomy is thought harmful [106,107].

## 9. Conclusions

A new thyroid neoplasm entity NIFTP, identified and discusses as a result of the diagnostic consensus of an international multidisciplinary expert panel group of pathologists, clinicians and patients, changed the field of thyroid diagnostics [5]. In 2017, the entity was implemented in a new edition of WHO classification of tumours of endocrine organs and was accepted by the American Thyroid Association [6,7]. According to the stricter and revised histopathological criteria from 2018, the diagnosis of NIFTP requires encapsulation or clear demarcation of the lesion with no vascular or capsular invasion, a follicular growth pattern with no well-formed papillae, no psammoma bodies and less than 30% of solid, trabecular, or insular growth pattern [12]. The original rate of 18.6% declined after the revised criteria were introduced. NIFTP molecular profile is more closely related to the follicular-pattern thyroid tumours with a predominant activating mutation of the *RAS* gene [33]; nevertheless, an NIFTP-specific molecular profile has not been established [45]. On the US level, NIFTP is observable as an oval, well-circumscribed nodule with well-detected margins and variable echogenicity [52,53]. New, emerging techniques such as elastography may have diagnostic potential. NIFTP is not a cytological diagnosis despite the fact that some cytomorphological differences were found. In the majority of cytological analyses, NIFTP cases were stratified in intermediate categories of TBSRTC, as follows: 29.8% in AUS/FLUS, 28.0% in FN and 21.2% SM categories. The 2nd edition of TBSRTC [95,97] revised the calculated ROMs, as NIFTP affects the ROM in the majority of TBSRTC categories.

## Data Availability

Not applicable.

## References

[1] Tallini G., Tuttle R.M., Ghossein R.A. (2017). The History of the Follicular Variant of Papillary Thyroid Carcinoma. J. Clin. Endocrinol. Metab..

[2] Lindsay S. (1960). Natural history of thyroid carcinoma. Ariz. Med..

[3] Chem K.T., Rosai J. (1977). Follicular variant of thyroid papillary carcinoma: A clinicopathologic study of six cases. Am. J. Surg. Pathol..

[4] Williams E.D. (2000). Guest Editorial: Two Proposals Regarding the Terminology of Thyroid Tumors. Int. J. Surg. Pathol..

[5] Nikiforov Y.E., Seethala R.R., Tallini G., Baloch Z.W., Basolo F., Thompson L.D., Barletta J.A., Wenig B.M., Al Ghuzlan A., Kakudo K. (2016). Nomenclature Revision for Encapsulated Follicular Variant of Papillary Thyroid Carcinoma: A Paradigm Shift to Reduce Overtreatment of Indolent Tumors. JAMA Oncol..

[6] Lloyd R.V., Osamura R.Y., Klöppel G., Rosai J. (2017). WHO Classification of Tumours of Endocrine Organs.

[7] Haugen B.R., Sawka A.M., Alexander E.K., Bible K.C., Caturegli P., Doherty G.M., Mandel S.J., Morris J.C., Nassar A., Pacini F. (2017). American Thyroid Association Guidelines on the Management of Thyroid Nodules and Differentiated Thyroid Cancer Task Force Review and Recommendation on the Proposed Renaming of Encapsulated Follicular Variant Papillary Thyroid Carcinoma Without Invasion to Noninvasive Follicular Thyroid Neoplasm with Papillary-Like Nuclear Features. Thyroid.

[8] Hodak S., Tuttle R.M., Maytal G., Nikiforov Y.E., Randolph G. (2016). Changing the Cancer Diagnosis: The Case of Follicular Variant of Papillary Thyroid Cancer-Primum Non Nocere and NIFTP. Thyroid.

[9] Parente D.N., Kluijfhout W.P., Bongers P.J., Verzijl R., Devon K.M., Rotstein L.E., Goldstein D.P., Asa S.L., Mete O., Pasternak J.D. (2018). Clinical Safety of Renaming Encapsulated Follicular Variant of Papillary Thyroid Carcinoma: Is NIFTP Truly Benign?. World J. Surg..

[10] Kim T.H., Lee M., Kwon A.Y., Choe J.H., Kim J.H., Kim J.S., Hahn S.Y., Shin J.H., Chung M.K., Son Y.I. (2018). Molecular genotyping of the non-invasive encapsulated follicular variant of papillary thyroid carcinoma. Histopathology.

[11] Cho U., Mete O., Kim M.H., Bae J.S., Jung C.K. (2017). Molecular correlates and rate of lymph node metastasis of non-invasive follicular thyroid neoplasm with papillary-like nuclear features and invasive follicular variant papillary thyroid carcinoma: The impact of rigid criteria to distinguish non-invasive follicular thyroid neoplasm with papillary-like nuclear features. Mod. Pathol..

[12] Nikiforov Y.E., Baloch Z.W., Hodak S.P., Giordano T.J., Lloyd R.V., Seethala R.R., Wenig B.M. (2018). Change in Diagnostic Criteria for Noninvasive Follicular Thyroid Neoplasm With Papillarylike Nuclear Features. JAMA Oncol..

[13] Alves V.A.F., Kakudo K., LiVolsi V., Lloyd R.V., Nikiforov Y.E., Nose V., Papotti M., Thompson L.D.R. (2018). Noninvasive Follicular Thyroid Neoplasm With Papillary-Like Nuclear Features (NIFTP): Achieving Better Agreement By Refining Diagnostic Criteria. Clinics.

[14] LiVolsi V.A., Baloch Z. (2021). Noninvasive Follicular Tumor with Papillary-like Nuclear Features: A Practice Changer in Thyroid Pathology. Arch. Pathol. Lab. Med..

[15] Seethala R.R., Baloch Z.W., Barletta J.A., Khanafshar E., Mete O., Sadow P.M., LiVolsi V.A., Nikiforov Y.E., Tallini G., Thompson L.D. (2018). Noninvasive follicular thyroid neoplasm with papillary-like nuclear features: A review for pathologists. Mod. Pathol..

[16] Fakhar Y., Khooei A., Aghaee A., Mohammadzadeh Kosari H., Wartofsky L., Zakavi S.R. (2021). Bone metastasis from noninvasive follicular thyroid neoplasm with papillary-like nuclear features (NIFTP); a case report. BMC Endocr. Disord..

[17] Rosario P.W., Mourao G.F. (2019). Noninvasive follicular thyroid neoplasm with papillary-like nuclear features (NIFTP): A review for clinicians. Endocr.-Relat. Cancer.

[18] Rana C., Vuong H.G., Nguyen T.Q., Nguyen H.C., Jung C.K., Kakudo K., Bychkov A. (2021). The Incidence of Noninvasive Follicular Thyroid Neoplasm with Papillary-Like Nuclear Features: A Meta-Analysis Assessing Worldwide Impact of the Reclassification. Thyroid.

[19] Liu Z., Bychkov A., Jung C.K., Hirokawa M., Sui S., Hong S., Lai C.R., Jain D., Canberk S., Kakudo K. (2019). Interobserver and intraobserver variation in the morphological evaluation of noninvasive follicular thyroid neoplasm with papillary-like nuclear features in Asian practice. Pathol. Int..

[20] Lloyd R.V., Erickson L.A., Casey M.B., Lam K.Y., Lohse C.M., Asa S.L., Chan J.K., DeLellis R.A., Harach H.R., Kakudo K. (2004). Observer variation in the diagnosis of follicular variant of papillary thyroid carcinoma. Am. J. Surg. Pathol..

[21] Elsheikh T.M., Asa S.L., Chan J.K., DeLellis R.A., Heffess C.S., LiVolsi V.A., Wenig B.M. (2008). Interobserver and intraobserver variation among experts in the diagnosis of thyroid follicular lesions with borderline nuclear features of papillary carcinoma. Am. J. Clin. Pathol..

[22] Jung C.K., Bychkov A., Song D.E., Kim J.H., Zhu Y., Liu Z., Keelawat S., Lai C.R., Hirokawa M., Kameyama K. (2021). Molecular Correlates and Nuclear Features of Encapsulated Follicular-Patterned Thyroid Neoplasms. Endocrinol. Metab..

[23] Bychkov A., Jung C.K., Liu Z., Kakudo K. (2018). Noninvasive Follicular Thyroid Neoplasm with Papillary-Like Nuclear Features in Asian Practice: Perspectives for Surgical Pathology and Cytopathology. Endocr. Pathol..

[24] Ruanpeng D., Cheungpasitporn W., Thongprayoon C., Hennessey J.V., Shrestha R.T. (2019). Systematic Review and Meta-analysis of the Impact of Noninvasive Follicular Thyroid Neoplasm with Papillary-Like Nuclear Features (NIFTP) on Cytological Diagnosis and Thyroid Cancer Prevalence. Endocr. Pathol..

[25] Cancer Genome Atlas Research N. (2014). Integrated genomic characterization of papillary thyroid carcinoma. Cell.

[26] Song Y.S., Park Y.J. (2020). Mechanisms of TERT Reactivation and Its Interaction with BRAFV600E. Endocrinol. Metab..

[27] Lee S.E., Hwang T.S., Choi Y.L., Kim W.Y., Han H.S., Lim S.D., Kim W.S., Yoo Y.B., Kim S.K. (2017). Molecular Profiling of Papillary Thyroid Carcinoma in Korea with a High Prevalence of BRAF(V600E) Mutation. Thyroid.

[28] Brandler T.C., Zhou F., Liu C.Z., Cho M., Lau R.P., Simsir A., Patel K.N., Sun W. (2017). Can noninvasive follicular thyroid neoplasm with papillary-like nuclear features be distinguished from classic papillary thyroid carcinoma and follicular adenomas by fine-needle aspiration?. Cancer Cytopathol..

[29] Strickland K.C., Eszlinger M., Paschke R., Angell T.E., Alexander E.K., Marqusee E., Nehs M.A., Jo V.Y., Lowe A., Vivero M. (2018). Molecular Testing of Nodules with a Suspicious or Malignant Cytologic Diagnosis in the Setting of Non-Invasive Follicular Thyroid Neoplasm with Papillary-Like Nuclear Features (NIFTP). Endocr. Pathol..

[30] Zhao L., Dias-Santagata D., Sadow P.M., Faquin W.C. (2017). Cytological, molecular, and clinical features of noninvasive follicular thyroid neoplasm with papillary-like nuclear features versus invasive forms of follicular variant of papillary thyroid carcinoma. Cancer Cytopathol..

[31] Paulson V.A., Shivdasani P., Angell T.E., Cibas E.S., Krane J.F., Lindeman N.I., Alexander E.K., Barletta J.A. (2017). Noninvasive Follicular Thyroid Neoplasm with Papillary-Like Nuclear Features Accounts for More Than Half of “Carcinomas” Harboring RAS Mutations. Thyroid.

[32] Pusztaszeri M., Bongiovanni M. (2019). The impact of non-invasive follicular thyroid neoplasm with papillary-like nuclear features (NIFTP) on the diagnosis of thyroid nodules. Gland Surg..

[33] Pool C., Walter V., Bann D., Goldenberg D., Broach J., Hennessy M., Cottrill E., Washburn E., Williams N., Crist H. (2019). Molecular characterization of tumors meeting diagnostic criteria for the non-invasive follicular thyroid neoplasm with papillary-like nuclear features (NIFTP). Virchows Arch..

[34] Zajkowska K., Kopczynski J., Gozdz S., Kowalska A. (2020). Noninvasive follicular thyroid neoplasm with papillary-like nuclear features: A problematic entity. Endocr. Connect..

[35] Brandler T.C., Yee J., Zhou F., Cho M., Cangiarella J., Wei X.J., Yee-Chang M., Sun W. (2018). Does noninvasive follicular thyroid neoplasm with papillary-like nuclear features have distinctive features on sonography?. Diagn. Cytopathol..

[36] Afkhami M., Karunamurthy A., Chiosea S., Nikiforova M.N., Seethala R., Nikiforov Y.E., Coyne C. (2016). Histopathologic and Clinical Characterization of Thyroid Tumors Carrying the BRAF(K601E) Mutation. Thyroid.

[37] Johnson D.N., Furtado L.V., Long B.C., Zhen C.J., Wurst M., Mujacic I., Kadri S., Segal J.P., Antic T., Cipriani N.A. (2018). Noninvasive Follicular Thyroid Neoplasms with Papillary-like Nuclear Features Are Genetically and Biologically Similar to Adenomatous Nodules and Distinct From Papillary Thyroid Carcinomas with Extensive Follicular Growth. Arch. Pathol. Lab. Med..

[38] Geramizadeh B., Maleki Z. (2019). Non-invasive follicular thyroid neoplasm with papillary-like nuclearfeatures (NIFTP): A review and update. Endocrine.

[39] Kim M.J., Won J.K., Jung K.C., Kim J.H., Cho S.W., Park D.J., Park Y.J. (2018). Clinical Characteristics of Subtypes of Follicular Variant Papillary Thyroid Carcinoma. Thyroid.

[40] Song Y.S., Won J.K., Yoo S.K., Jung K.C., Kim M.J., Kim S.J., Cho S.W., Lee K.E., Yi K.H., Seo J.S. (2018). Comprehensive Transcriptomic and Genomic Profiling of Subtypes of Follicular Variant of Papillary Thyroid Carcinoma. Thyroid.

[41] Melo M., da Rocha A.G., Vinagre J., Batista R., Peixoto J., Tavares C., Celestino R., Almeida A., Salgado C., Eloy C. (2014). TERT promoter mutations are a major indicator of poor outcome in differentiated thyroid carcinomas. J. Clin. Endocrinol. Metab..

[42] Kuchareczko A., Kopczynski J., Kowalik A., Hincza K., Plusa A., Gozdz S., Kowalska A. (2021). Are molecular tests necessary to diagnose NIFTP?. Genes Cancer.

[43] Sohn S.Y., Lee J.J., Lee J.H. (2020). Molecular Profile and Clinicopathologic Features of Follicular Variant Papillary Thyroid Carcinoma. Pathol. Oncol. Res..

[44] Amendoeira I., Maia T., Sobrinho-Simoes M. (2018). Non-invasive follicular thyroid neoplasm with papillary-like nuclear features (NIFTP): Impact on the reclassification of thyroid nodules. Endocr.-Relat. Cancer.

[45] Paniza A.C.J., Mendes T.B., Viana M.D.B., Thomaz D.M.D., Chiappini P.B.O., Colozza-Gama G.A., Lindsey S.C., de Carvalho M.B., Alves V.A.F., Curioni O. (2019). Revised criteria for diagnosis of NIFTP reveals a better correlation with tumor biological behavior. Endocr. Connect..

[46] Jahanbani I., Al-Abdallah A., Ali R.H., Al-Brahim N., Mojiminiyi O. (2018). Discriminatory miRNAs for the Management of Papillary Thyroid Carcinoma and Noninvasive Follicular Thyroid Neoplasms with Papillary-Like Nuclear Features. Thyroid.

[47] Park J.L., Kim S.K., Jeon S., Jung C.K., Kim Y.S. (2021). MicroRNA Profile for Diagnostic and Prognostic Biomarkers in Thyroid Cancer. Cancers.

[48] Borrelli N., Denaro M., Ugolini C., Poma A.M., Miccoli M., Vitti P., Miccoli P., Basolo F. (2017). miRNA expression profiling of ‘noninvasive follicular thyroid neoplasms with papillary-like nuclear features’ compared with adenomas and infiltrative follicular variants of papillary thyroid carcinomas. Mod. Pathol..

[49] Kalfert D., Ludvikova M., Pesta M., Ludvik J., Dostalova L., Kholova I. (2020). Multifunctional Roles of miR-34a in Cancer: A Review with the Emphasis on Head and Neck Squamous Cell Carcinoma and Thyroid Cancer with Clinical Implications. Diagnostics.

[50] Ludvikova M., Kalfert D., Kholova I. (2015). Pathobiology of MicroRNAs and Their Emerging Role in Thyroid Fine-Needle Aspiration. Acta Cytol..

[51] Mahmoudian-Sani M.R., Jalali A., Jamshidi M., Moridi H., Alghasi A., Shojaeian A., Mobini G.R. (2019). Long Non-Coding RNAs in Thyroid Cancer: Implications for Pathogenesis, Diagnosis, and Therapy. Oncol. Res. Treat..

[52] Kwon M.R., Shin J.H., Hahn S.Y., Oh Y.L., Kwak J.Y., Lee E., Lim Y. (2018). Histogram analysis of greyscale sonograms to differentiate between the subtypes of follicular variant of papillary thyroid cancer. Clin. Radiol..

[53] Yang G.C.H., Fried K.O. (2018). Pathologic basis of the sonographic differences between thyroid cancer and noninvasive follicular thyroid neoplasm with papillary-like nuclear features. Ultrasonography.

[54] Hoang J.K., Middleton W.D., Farjat A.E., Langer J.E., Reading C.C., Teefey S.A., Abinanti N., Boschini F.J., Bronner A.J., Dahiya N. (2018). Reduction in Thyroid Nodule Biopsies and Improved Accuracy with American College of Radiology Thyroid Imaging Reporting and Data System. Radiology.

[55] Watkins L., O’Neill G., Young D., McArthur C. (2021). Comparison of British Thyroid Association, American College of Radiology TIRADS and Artificial Intelligence TIRADS with histological correlation: Diagnostic performance for predicting thyroid malignancy and unnecessary fine needle aspiration rate. Br. J. Radiol..

[56] Tessler F.N., Middleton W.D., Grant E.G. (2018). Thyroid Imaging Reporting and Data System (TI-RADS): A User’s Guide. Radiology.

[57] Tessler F.N., Middleton W.D., Grant E.G., Hoang J.K., Berland L.L., Teefey S.A., Cronan J.J., Beland M.D., Desser T.S., Frates M.C. (2017). ACR Thyroid Imaging, Reporting and Data System (TI-RADS): White Paper of the ACR TI-RADS Committee. J. Am. Coll. Radiol..

[58] Shin J.H., Baek J.H., Chung J., Ha E.J., Kim J.H., Lee Y.H., Lim H.K., Moon W.J., Na D.G., Park J.S. (2016). Ultrasonography Diagnosis and Imaging-Based Management of Thyroid Nodules: Revised Korean Society of Thyroid Radiology Consensus Statement and Recommendations. Korean J. Radiol..

[59] Lauria Pantano A., Maddaloni E., Briganti S.I., Beretta Anguissola G., Perrella E., Taffon C., Palermo A., Pozzilli P., Manfrini S., Crescenzi A. (2018). Differences between ATA, AACE/ACE/AME and ACR TI-RADS ultrasound classifications performance in identifying cytological high-risk thyroid nodules. Eur. J. Endocrinol..

[60] Middleton W.D., Teefey S.A., Reading C.C., Langer J.E., Beland M.D., Szabunio M.M., Desser T.S. (2018). Comparison of Performance Characteristics of American College of Radiology TI-RADS, Korean Society of Thyroid Radiology TIRADS, and American Thyroid Association Guidelines. AJR Am. J. Roentgenol..

[61] Kwon H., Jeon M.J., Yoon J.H., Hong S.J., Lee J.H., Kim T.Y., Shong Y.K., Kim W.B., Kim W.G., Song D.E. (2017). Preoperative clinicopathological characteristics of patients with solitary encapsulated follicular variants of papillary thyroid carcinomas. J. Surg. Oncol..

[62] Yang G.C.H., Fried K.O., Scognamiglio T. (2017). Sonographic and cytologic differences of NIFTP from infiltrative or invasive encapsulated follicular variant of papillary thyroid carcinoma: A Review of 179 Cases. Diagn. Cytopathol..

[63] Yang G.C.H., Fried K.O., Scognamiglio T. (2020). Can cytology and the Thyroid Imaging, Reporting, and Data System (TI-RADS) identify noninvasive follicular thyroid neoplasm with papillary-like nuclear features (NIFTP) before surgery?. J. Am. Soc. Cytopathol..

[64] Hahn S.Y., Shin J.H., Lim H.K., Jung S.L., Oh Y.L., Choi I.H., Jung C.K. (2017). Preoperative differentiation between noninvasive follicular thyroid neoplasm with papillary-like nuclear features (NIFTP) and non-NIFTP. Clin. Endocrinol..

[65] Lee H.S., Lee J.W., Park J.H., Kim W.S., Han H.S., Lee S.E. (2019). Comprehensive analysis for diagnosis of preoperative non-invasive follicular thyroid neoplasm with papillary-like nuclear features. PLoS ONE.

[66] Hahn S.Y., Shin J.H., Oh Y.L., Kim T.H., Lim Y., Choi J.S. (2017). Role of Ultrasound in Predicting Tumor Invasiveness in Follicular Variant of Papillary Thyroid Carcinoma. Thyroid.

[67] Boursier L., Clerc Urmes I., Garon J., Klein M., Demarquet L. (2020). Ultrasound and cytological characteristics of non-invasive follicular thyroid neoplasm with papillary-like nuclear features compared to papillary carcinomas. Ann. Endocrinol..

[68] Rosario P.W. (2018). Is Doppler ultrasonography of value for the differentiation between noninvasive follicular thyroid neoplasm with papillary-like nuclear features (NIFTP) and invasive encapsulated follicular variant of papillary thyroid carcinoma?. Clin. Endocrinol..

[69] You S.H., Lee K.E., Yoo R.E., Choi H.J., Jung K.C., Won J.K., Kang K.M., Yoon T.J., Choi S.H., Sohn C.H. (2018). Prevention of total thyroidectomy in noninvasive follicular thyroid neoplasm with papillary-like nuclear features (NIFTP) based on combined interpretation of ultrasonographic and cytopathologic results. Clin. Endocrinol..

[70] Figge J.J., Gooding W.E., Steward D.L., Yip L., Sippel R.S., Yang S.P., Scheri R.P., Sipos J.A., Mandel S.J., Mayson S.E. (2021). Do Ultrasound Patterns and Clinical Parameters Inform the Probability of Thyroid Cancer Predicted by Molecular Testing in Nodules with Indeterminate Cytology?. Thyroid.

[71] Trimboli P., Castellana M., Virili C., Havre R.F., Bini F., Marinozzi F., D’Ambrosio F., Giorgino F., Giovanella L., Prosch H. (2020). Performance of contrast-enhanced ultrasound (CEUS) in assessing thyroid nodules: A systematic review and meta-analysis using histological standard of reference. Radiol. Med..

[72] Zhao C.K., Xu H.X. (2019). Ultrasound elastography of the thyroid: Principles and current status. Ultrasonography.

[73] Garino F., Deandrea M., Motta M., Mormile A., Ragazzoni F., Palestini N., Freddi M., Gasparri G., Sgotto E., Pacchioni D. (2015). Diagnostic performance of elastography in cytologically indeterminate thyroid nodules. Endocrine.

[74] Stoian D., Borcan F., Petre I., Mozos I., Varcus F., Ivan V., Cioca A., Apostol A., Dehelean C.A. (2019). Strain Elastography as a Valuable Diagnosis Tool in Intermediate Cytology (Bethesda III) Thyroid Nodules. Diagnostics.

[75] Borlea A., Stoian D., Cotoi L., Sporea I., Lazar F., Mozos I. (2020). Thyroid Multimodal Ultrasound Evaluation—Impact on Presurgical Diagnosis of Intermediate Cytology Cases. Appl. Sci..

[76] Mahajan S., Agarwal S., Kocheri N., Jain D., Mathur S.R., Iyer V.K. (2018). Cytopathology of non-invasive follicular thyroid neoplasm with papillary-like nuclear features: A comparative study with similar patterned papillary thyroid carcinoma variants. Cytopathology.

[77] Maletta F., Massa F., Torregrossa L., Duregon E., Casadei G.P., Basolo F., Tallini G., Volante M., Nikiforov Y.E., Papotti M. (2016). Cytological features of “noninvasive follicular thyroid neoplasm with papillary-like nuclear features” and their correlation with tumor histology. Hum. Pathol..

[78] Chandler J.B., Colunga M., Prasad M.L., Callender G.G., Quinn C., Chhieng D., Adeniran A.J. (2017). Identification of distinct cytomorphologic features in the diagnosis of NIFTP at the time of preoperative FNA: Implications for patient management. Cancer Cytopathol..

[79] Diaz Del Arco C., Fernandez Acenero M.J. (2018). Noninvasive Follicular Thyroid Neoplasm with Papillary-Like Nuclear Features: Can Cytology Face the Challenge of Diagnosis in the Light of the New Classification?. Acta Cytol..

[80] Hirokawa M., Higuchi M., Suzuki A., Hayashi T., Kuma S., Miyauchi A. (2017). Noninvasive follicular thyroid neoplasm with papillary-like nuclear features: A single-institutional experience in Japan. Endocr. J..

[81] Hirokawa M., Higuchi M., Suzuki A., Hayashi T., Kuma S., Miyauchi A. (2020). Prevalence and diagnostic significance of noninvasive follicular thyroid neoplasm with papillary-like nuclear features among tumors previously diagnosed as follicular adenoma: A single-institutional study in Japan. Endocr. J..

[82] Howitt B.E., Chang S., Eszlinger M., Paschke R., Drage M.G., Krane J.F., Barletta J.A. (2015). Fine-needle aspiration diagnoses of noninvasive follicular variant of papillary thyroid carcinoma. Am. J. Clin. Pathol..

[83] Jaconi M., Manzoni M., Pincelli A.I., Giardini V., Scardilli M., Smith A., Fellegara G., Pagni F. (2017). The impact of the non-invasive follicular thyroid neoplasm with papillary-like nuclear feature terminology in the routine diagnosis of thyroid tumours. Cytopathology.

[84] Koshikawa T., Fujita N., Ueda N., Ota Y., Sasaki E., Murakami Y., Hosoda W., Yatabe Y., Hanai N., Higuchi M. (2019). Important cytological findings for distinction between follicular variant and conventional papillary thyroid carcinoma, including noninvasive follicular thyroid tumors with papillary-like nuclear features. Endocr. J..

[85] Legesse T., Parker L., Heath J., Staats P.N. (2019). Distinguishing non-invasive follicular thyroid neoplasm with papillary-like nuclear features (NIFTP) from classic and invasive follicular-variant papillary thyroid carcinomas based on cytologic features. J. Am. Soc. Cytopathol..

[86] Maleki S., Zandvakili A., Gera S., Khutti S.D., Gersten A., Khader S.N. (2019). Differentiating Noninvasive Follicular Thyroid Neoplasm with Papillary-Like Nuclear Features from Classic Papillary Thyroid Carcinoma: Analysis of Cytomorphologic Descriptions Using a Novel Machine-Learning Approach. J. Pathol. Inform..

[87] Selvaggi S.M. (2019). The presence of multinucleated giant cells: Noninvasive follicular thyroid neoplasm with papillary-like nuclear features vs the follicular variant of papillary thyroid carcinoma. Diagn. Cytopathol..

[88] Strickland K.C., Howitt B.E., Marqusee E., Alexander E.K., Cibas E.S., Krane J.F., Barletta J.A. (2015). The Impact of Noninvasive Follicular Variant of Papillary Thyroid Carcinoma on Rates of Malignancy for Fine-Needle Aspiration Diagnostic Categories. Thyroid.

[89] Strickland K.C., Vivero M., Jo V.Y., Lowe A.C., Hollowell M., Qian X., Wieczorek T.J., French C.A., Teot L.A., Sadow P.M. (2016). Preoperative Cytologic Diagnosis of Noninvasive Follicular Thyroid Neoplasm with Papillary-Like Nuclear Features: A Prospective Analysis. Thyroid.

[90] Yan L., Sethi S., Park J.W. (2019). Cytologic and clinical features of NIFTP: Can we diagnose based on preoperative fine-needle aspiration. Diagn. Cytopathol..

[91] Zhang Z., Chhieng D., Harshan M., Zheng X., Zakowski M. (2019). Cytological features of noninvasive follicular thyroid neoplasm with papillary-like nuclear features (NIFTP). J. Am. Soc. Cytopathol..

[92] Bizzarro T., Martini M., Capodimonti S., Straccia P., Lombardi C.P., Pontecorvi A., Larocca L.M., Rossi E.D. (2016). Young investigator challenge: The morphologic analysis of noninvasive follicular thyroid neoplasm with papillary-like nuclear features on liquid-based cytology: Some insights into their identification. Cancer Cytopathol..

[93] Haaga E., Kalfert D., Ludvikova M., Kholova I. (2021). Non-Invasive Follicular Thyroid Neoplasm with Papillary-Like Nuclear Features Is Not a Cytological Diagnosis, but It Influences Cytological Diagnosis Outcomes: A Systematic Review and Meta-Analysis. Acta Cytol..

[94] Ali S.Z., Cibas E.S. (2010). The Bethesda System for Reporting Thyroid Cytopathology: Definitions, Criteria and Explanatory Notes.

[95] Ali S.Z., Cibas E.S. (2018). The Bethesda System for Reporting Thyroid Cytopathology.

[96] Bongiovanni M., Giovanella L., Romanelli F., Trimboli P. (2019). Cytological Diagnoses Associated with Noninvasive Follicular Thyroid Neoplasms with Papillary-Like Nuclear Features According to the Bethesda System for Reporting Thyroid Cytopathology: A Systematic Review and Meta-Analysis. Thyroid.

[97] Faquin W.C., Wong L.Q., Afrogheh A.H., Ali S.Z., Bishop J.A., Bongiovanni M., Pusztaszeri M.P., VandenBussche C.J., Gourmaud J., Vaickus L.J. (2016). Impact of reclassifying noninvasive follicular variant of papillary thyroid carcinoma on the risk of malignancy in The Bethesda System for Reporting Thyroid Cytopathology. Cancer Cytopathol..

[98] Shrestha R.T., Ruanpeng D., Hennessey J.V. (2019). Cytomorphology of Noninvasive Follicular Thyroid Neoplasm with Papillary-Like Nuclear Features and the Impact of New Nomenclature on Molecular Testing. Med. Sci..

[99] Baloch Z.W., Seethala R.R., Faquin W.C., Papotti M.G., Basolo F., Fadda G., Randolph G.W., Hodak S.P., Nikiforov Y.E., Mandel S.J. (2016). Noninvasive follicular thyroid neoplasm with papillary-like nuclear features (NIFTP): A changing paradigm in thyroid surgical pathology and implications for thyroid cytopathology. Cancer Cytopathol..

[100] Lau R.P., Paulsen J.D., Brandler T.C., Liu C.Z., Simsir A., Zhou F. (2017). Impact of the Reclassification of “Noninvasive Encapsulated Follicular Variant of Papillary Thyroid Carcinoma” to “Noninvasive Follicular Thyroid Neoplasm with Papillary-Like Nuclear Features” on the Bethesda System for Reporting Thyroid Cytopathology: A Large Academic Institution’s Experience. Am. J. Clin. Pathol..

[101] Layfield L.J., Baloch Z.W., Esebua M., Kannuswamy R., Schmidt R.L. (2017). Impact of the Reclassification of the Non-Invasive Follicular Variant of Papillary Carcinoma as Benign on the Malignancy Risk of the Bethesda System for Reporting Thyroid Cytopathology: A Meta-Analysis Study. Acta Cytol..

[102] Bongiovanni M., Faquin W.C., Giovanella L., Durante C., Kopp P., Trimboli P. (2019). Impact of non-invasive follicular thyroid neoplasms with papillary-like nuclear features (NIFTP) on risk of malignancy in patients undergoing lobectomy/thyroidectomy for suspected malignancy or malignant fine-needle aspiration cytology findings: A systematic review and meta-analysis. Eur. J. Endocrinol..

[103] Chereau N., Greilsamer T., Mirallié E., Sadowski S.M., Pusztaszeri M., Triponez F., Baud G., Pattou F., Christou N., Mathonnet M. (2019). NIFT-P: Are they indolent tumors? Results of a multi-institutional study. Surgery.

[104] Mao M.L., Joyal T., Picado O., Kerr D., Lew J.I., Farrá J.C. (2018). Noninvasive follicular thyroid neoplasm with papillary-like nuclear features reclassification and its impact on thyroid malignancy rate and treatment. J. Surg. Res..

[105] Kiernan C.M., Weiss V.L., Mehrad M., Ely K., Baregamian N., Solórzano C.C. (2018). New terminology-noninvasive follicular neoplasm with papillary-like nuclear features (NIFTP) and its effect on the rate of malignancy at a single institution. Surgery.

[106] Kakudo K. (2018). How to handle borderline/precursor thyroid tumors in management of patients with thyroid nodules. Gland Surg..

[107] Kakudo K. (2017). Unsettled issues in Non-invasive encapsulated/well circumscribed follicular thyroid neoplasm with papillary-like nuclear features (NIFTP) and precursor thyroid tumors. J. Basic Clin. Med..

